# A Comparative Study on Clinical Evaluation of the Hypolipidemic Effects of Allium sativum, Trigonella foenum-graecum, Commiphora mukul, Picrorhiza kurroa, and Piper nigrum: A Pilot Study

**DOI:** 10.7759/cureus.26597

**Published:** 2022-07-05

**Authors:** Rumana F Shaikh, Mohammed Taher Ali, Ashfaq A Mohsin, Sanket D Hiware, Arafat Ahmad, Syed Rehan H Daimi, Khwaja Moizuddin, Siraj A Shaikh, Faiza B Siddiqui

**Affiliations:** 1 Pharmacology, Imam Abdulrahman Bin Faisal University, Dammam, SAU; 2 Clinical Pharmacology, Imam Abdulrahman Bin Faisal University, Dammam, SAU; 3 Pharmaceutics, Imam Abdulrahman Bin Faisal University, Dammam, SAU; 4 Anatomy, Imam Abdulrahman Bin Faisal University, Dammam, SAU; 5 Biochemistry, Imam Abdulrahman Bin Faisal University, Dammam, SAU

**Keywords:** hmg-coa reductase inhibitors, picrorhiza kurroa, hyperlipidemia, trigonella foenum graecum, commiphora mukul, allium sativum, serum triglycerides, total cholesterol, electrocardiogram

## Abstract

Background

Cardiovascular disease is a leading cause of morbidity and mortality. Therefore, it is essential to prevent cardiovascular diseases by correcting modifiable risk factors such as lowering lipid levels, lowering blood pressure, improving eating habits, giving up smoking, etc. The present study assessed the efficacy of herbal preparation containing *Allium sativum (A. sativum*), *Commiphora mukul (C. mukul)*, and *Trigonella foenum*-*graecum* (*T. foenum-graecum*) in patients with hyperlipidemia.

Methodology

Patients were given extracts of *A. sativum* 350 mg, *T. foenum-graecum* 350 mg, *C. mukul* 200 mg, Picrorhiza kurroa (*P. kurroa*) 200 mg, and Piper nigrum (*P. nigrum*) 5 mg. Unichem Laboratories, Mumbai, provided placebo tablets similar in shape and size to herbal tablets. Patients were assessed for compliance, and a complete lipid profile was done at DO, D15, D46, D76, and D106. In addition, total cholesterol and high-density lipoprotein-cholesterol (HDL-C) serum triglyceride were estimated by the respective methods throughout the study.

Results

The weight of the patients remained stable, the mean weight before being 65.42 ± 8.35 kg and after completion of the study being 65.42 ± 8.35 kg. There were no changes in the ECG during or after the drug therapy in any of the patients. Group A comprised nine patients, and group B had ten patients. Serum creatinine (mg %) was 0.94 and 0.95, fasting blood sugar mg (%) was 111.05 and 99.63, and postprandial blood sugar (mg %) was 150.89 and 147.94 on pre-treatment and post-treatment, respectively. The mean serum triglyceride levels in group A were 271.11, 261.11, 293.89, 167.22, and 128.89, and serum HDL- C levels were 46.11, 46.11, 54.44, 52.22, and 54.44. Serum triglyceride levels in group B were 268, 268.5, 202, 171, and 116, and serum HDL- C levels were 48.5, 48, 50, 50, and 53.5 on day 0, 15, 46, 76, and 106, respectively. A significant reduction in total cholesterol levels was observed on D46, D76, and D106, with a maximum reduction on D76 (25.36%). Similarly, a reduction in serum triglyceride was also observed on D46, D76, and D106, with a maximum reduction on D106 (52.02%). A significant difference was observed (P <0.05). There was also a significant reduction of low-density lipoprotein cholesterol (*LDL*-*C*) on D46, D76, and D106, with the maximum reduction on D76 (28.79%). There was a significant rise of HDL-C on D46 and D106, with a maximum rise on D106 (15.41%). A significant difference was observed (P <0.05).

Conclusion

The study drugs are safe and efficacious in reducing the total cholesterol, serum triglycerides, LDL-C levels, and increasing HDL-C levels.

## Introduction

Cardiovascular disease is a leading cause of morbidity and mortality in western society and is rapidly becoming the leading cause of death worldwide. Large-scale epidemiological studies and reviews of the literature over the last 30 years have identified several new risk factors for the development of cardiovascular disease [1]. Many traditional risk factors for coronary artery diseases (CAD), such as advanced age, male gender, obesity, diabetes, physical inactivity, elevated blood pressure, and smoking, can be identified during a routine physical examination and medical history [2]. However, other markers for increased cardiovascular risk can be detected only by careful laboratory evaluation. Of these, the best known is elevated serum lipids, particularly elevated low-density lipoprotein cholesterol (LDL-C) and triglycerides [3].

Thus, there is a positive relationship between elevated total cholesterol, triglycerides, and LDL-C and the development of CAD. On the other hand, the high-density lipoprotein cholesterol (HDL-C) appeared to have an inverse relation to the risk of coronary heart disease. A recent meta-analysis has indicated that for each 1 mmol increase in plasma triglyceride, there is approximately a 32% increase in coronary disease risk for men and a 76% increase in women [2]. Thus hypertriglyceridemia and hypercholesterolemia are independent risk factors for CAD.

Many epidemiologic and clinical studies have shown that lipid-lowering treatment that decreases LDL-C by more than 20% decreases the severity of coronary atherosclerosis and the incidence of clinical events like myocardial infarction, stroke, and death. So the focus of attention has been shifted to lipid-lowering agents, but so far, the lipid-lowering agents available are not ideal. Hence, there is a strong need to evaluate newer hypolipidemic agents with better therapeutic index and improved cost-effectiveness.

The advent of the β-hydroxy β-methylglutaryl-CoA (HMG-CoA) reductase inhibitors or "statins" has revolutionized the pharmacologic treatment of dyslipidemia. Though they are very efficacious and well-tolerated, they are not devoid of serious side effects like hepatotoxicity, myositis, rhabdomyolysis, etc. Although these agents appear more promising, the high cost is a limiting factor, especially in a developing country like India. Therefore, it is necessary to search for new agents that are equally effective but more economical.

Hyperlipidemia may occur because of a primarily genetic disorder or secondary to conditions like hypothyroidism, diabetes mellitus, nephrotic syndrome, obesity, etc. Therefore, it becomes essential to prevent cardiovascular diseases by correcting modifiable risk factors such as lowering lipid levels, lowering blood pressure, improving eating habits, giving up smoking, etc [4]. Thus, preventive medical therapies and lifestyle changes can reduce cardiovascular mortality and the need for expensive revascularization procedures. The studies carried out so far have used either of these plants. However, the effects of these plants in combination have never been explored [5]. Therefore, a study was planned to evaluate the efficacy of herbal preparation containing *Allium sativum* (*A. sativum*), *Commiphora mukul* (*C. mukul*), and *Trigonella foenum-graecum* (*T. foenum-graecum*) in patients with hyperlipidemia.

## Materials and methods

The patients attending the hypertension clinic of King Edward Memorial (KEM) Hospital after they satisfied the inclusion and exclusion criteria were selected for the study. Protocol prepared for the unmatched study was submitted to the institutional ethics committee of King Edward Memorial Hospital and Seth Gordhandas Sunderdas Medical College, Mumbai, with vide letter no. "IRB-1998-01-118" and the study was initiated after getting the approval and written informed consent of patients.

The patients of either sex, above 18 years of age, with raised serum cholesterol levels (greater than 240 mg % and/or raised triglyceride levels (greater than 250 mg %), willing to come for regular follow-up visits, willing to take medications as directed, and who has given informed consent were included in the study. However, pregnant women, nursing mothers, women of childbearing potential, those who do not follow adequate contraception measures, and patients with significant atherosclerotic disease, including patients with significant CAD, were excluded from the study. In addition, patients with a history of acute myocardial infarction in the preceding six months, uncontrolled/poorly controlled diabetes mellitus or juvenile diabetes mellitus or any endocrine disorders, patients with multiple risk factors for CAD, prior therapy with any lipid-lowering agents, including statins within preceding four weeks, and any secondary cause of hyperlipidemia were also excluded from the study. Also, people with a history of smoking, alcohol, and/or drug abuse, people with severe hepatic, renal or cardiac dysfunction or uncontrolled hypertension, treatment with any investigational drug in preceding four weeks, familial hypercholesterolemia and hyperlipoproteinemia (type III) as there is an increased risk of CAD, and patients unwilling to take regular medication and/or come for follow-up were also excluded from the study.

After selection, the patients were given two placebo tablets twice a day for 14 days. The herbal product containing extracts of *A. sativum* 350 mg, *T. foenum-graecum* 350 mg, *C. mukul* 200 mg, *P. kurroa* 200 mg, and *P. nigrum* 5 mg were used in the study. The Unichem Laboratories, Mumbai, provided placebo tablets similar in shape and size as that to herbal tablets. On the 15th day, patients were assessed for compliance, and then the study drug was prescribed in the dose of two tablets twice a day with meals for three months. A detailed medical history and physical examination were carried out on all patients on day 0, day 15, day 46, day 76, and day 106. Blood was collected for hematological (hemoglobin, total and differential WBC count), sugar estimation, and routine organ function tests (serum bilirubin, serum glutamic-pyruvic transaminase [SGPT], blood urea nitrogen [BUN], and serum creatinine) before starting the therapy (i.e., DO) and at the end of the study (i.e., D106). ECG monitoring was done on DO, D46, and D106. Patients were assessed for compliance, and a complete lipid profile was done at DO, D15, D46, D76, and D106. The respective methods throughout the study estimated total cholesterol and HDL-C serum triglyceride.

The efficacy of the study drug was assessed by estimating the serum lipid levels at periodic intervals. The efficacy was established if there was at least a 20% fall in serum cholesterol and/or serum triglyceride or a rise of 20% in HDL-C within three months. Antihypertensive drugs like β-blockers, ACE-inhibitors, thiazides, calcium channel blockers, antidiabetic drugs like glibenclamide, tolbutamide, and antianginal drugs like aspirin, Sorbitrate (used prophylactically) were continued during the study. All observed or volunteered adverse events during the study period were recorded, and they were treated appropriately.

Sample size estimation

This is the first study to explore the feasibility and lipid-improving effect and safety of the above-mentioned combinations of the study drugs compared with placebo granules. Therefore, a formal sample size calculation is not required [6]. However, based on a study recommending a minimum of eight participants per group for a pilot study and considering an 80% upper confidence limit and 90% powered the main trial, we selected 9 and 10 participants per group as a sample size for this trial [7].

Statistical analysis

The data were analyzed using Statistical Package for Social Sciences (SPSS) version 21. The student t-test was done on all parametric data, whereas the Chi-squared test was performed on non-parametric data. P <0.05 was considered statistically significant.

## Results

All patients successfully participated in the study, and no dropouts were found. The study revealed that no significant difference was obtained in comparing the hemoglobin and WBC count of the patients at pre (Day 0) and post-treatment (Day 106) period (p>0.05), except for monocyte levels (p=0.001). On Day 0, serum creatinine (mg %) was 0.94 ± 0.09, and on Day 106, it was found to be 0.95 ± 0.06 (p=0.98). However, significant results were obtained comparing fasting blood sugar levels between the pre-treatment and post-treatment groups (p=0.01). In the pre-treatment group, the postprandial blood sugar was 150.89 ± 57.49, while in the post-treatment group, it was observed to be 147.94 ± 38.70 (p=0.75) (Table 1).

**Table 1:** Pre- and post-treatment values. BUN: Blood urea nitrogen; SGPT: Serum glutamic pyruvic transaminase.

Parameter	Pre-treatment (Day 0)	Post-treatment (Day 106)	P-value
Hemoglobin (gm %)	12.44 ± 1.4	12.51 ± 1.11	0.78
WBC count cells per cu. mm.	7594.7 ± 1633.4	7431.5 ± 884.46	0.58
Differential count			
Polymorphs %	58.84 ± 6.39	59.57 ± 4.63	0.63
Lymphocytes %	34.73 ± 8.69	34 ± 7.85	0.62
Monocytes %	0.63 ± 1.01	0.1 ± 0.45	0.001
Eosinophils %	1.26 ± 1.28	1.26 ± 0.65	0.99
Basophils %	0	0	-
SGPT (units per litre)	15.73 ± 5.24	16.31 ± 4.78	0.56
BUN mg %	11.45 ± 3.15	11.37 ± 2.67	0.89
Serum creatinine mg %	0.94 ± 0.09	0.95 ± 0.06	0.98
Fasting blood sugar mg %	111.05 ± 39.96	99.63 ± 22.56	0.01
Postprandial blood sugar mg %	150.89 ± 57.49	147.94 ± 38.70	0.75

A reduction in total cholesterol levels was observed on D46, D76, and D106, with the maximum reduction on D76 (25.36%) (p=0.0001). Similarly, a reduction in serum triglyceride was also observed on D46, D76, and D106, with a maximum reduction on D106 (52.02%) (p=0.0001). There was also a significant reduction of LDL-C on D46, D76, and D106, with a maximum and statistically significant reduction on D76 (28.79%) (P=0.0001). Conversely, there was a significant rise of HDL-C on D46 and D106, with a maximum rise on D106 (15.41%) (p=0.0001). The total cholesterol to HDL-C ratio, a predictor of risk, also significantly reduced from 6.3 ±1.36 to 4.39 ± 1.02 at the end of the study period (p=0.02). Similarly, the LDL-C to HDL-C ratio significantly reduced from 4.15 ± 1.29 to 2.96 ± 1.00 at the end of the study (p=0.001) (Table 2). 

**Table 2:** Plasma lipid profile (mg %) of patients treated with the study drug. *p<0.05 LDL-C: Low-density lipoprotein-cholesterol; HDL-C: High-density lipoprotein-cholesterol; TC: Total cholesterol.

Parameter	Day 0	Day 15	Day 46	Day 76	Day 106	P-value (between Day 15 to Day 106)
Cholesterol (mg %)	293.42 ± 43.62	291.58 ± 45.06	251.84 ± 28.83*	239.71 ± 22.54*	228.42 ± 33.67*	0.0001*
% decrease	--	--	12.42 ± 12.66	25.36 ± 10.21	20.14 ± 15.64	
Triglyceride	269.47 ± 52.41	265 ± 46.19	198.16 ± 52.18*	169.21 ± 57.72*	122.10 ± 43.98*	0.0001*
% decrease	--	--	24.62 ± 16.08	33.57 ± 25.20	52.02 ± 20.04	
LDL-C	192.16 ± 48.01	190.95 ± 49.67	159.58 ± 27.69*	129.58 ± 27.46*	151.63 ± 34.59*	0.0001*
% decrease	--	--	12.59 ± 23.05	28.79 ± 20.84	15.48 ± 28.42	
HDL-C	47.37 ± 5.36	47.11 ± 4.81	52.63 ± 8.23*	51.05 ± 11.25	53.95 ± 11.37*	0.02*
% increase	--	--	12.91 ± 21.69	9.82 ± 28.98	15.41 ± 26.85	
TC: HDL-C ratio	6.3 ± 1.36	6.25 ± 1.28	4.85 ± 0.72*	4.39 ± 1.15*	4.39 ± 1.02*	0.0001*
LDL-C: HDL-C ratio	4.15 ± 1.29	4.07 ± 1.33	3.07 ± 0.67*	2.73 ± 1.12*	2.96 ± 1.001*	0.001*

Drugs like β-blockers and thiazides are known to increase triglyceride levels and lower HDL-C levels. Out of the 19 patients, nine patients who were on these drugs were grouped separately as Group A (n=9), and the data was analyzed and compared with the other group (n=10) who were on other antihypertensive drugs. There was a significant reduction in serum triglyceride on D106 and an increase in HDL-C at the end as compared to D15 in each group. The comparison of the two groups is presented in Table 3.

**Table 3:** Comparison of serum triglyceride and HDL-C levels in Groups A and B. *p<0.05 HDL-C: High-density lipoprotein-cholesterol.

Days	Group A (n = 9)		Group B (n = 10)	
	Serum triglyceride	Serum HDL-C	Serum triglyceride	Serum HDL-C
Day 0	271.11 ± 54.82	46.11 ± 4.85	268 ± 53.08	48.5 ± 5.79
Day 15	261.11 ± 43.64	46.11 ± 4.16	268.5 ± 50.44	48 ± 5.37
Day 46	193.89 ± 46.22	54.44 ± 8.46	202 ± 59.26	50 ± 5.43
Day 76	167.22 ± 60.42	52.22 ± 6.94	171 ± 58.39	50 ± 7.82
Day 106	128.89 ± 56.17	54.44 ± 7.01	116 ± 31.25	53.5 ± 6.14
P-value (Day 15 and Day 106)	0.0001*	0.007*	0.0001*	0.04*

## Discussion

The present study evaluated the efficacy of herbal preparation containing *A. sativum, C. mukul, and T. foenum graecum* in patients with hyperlipidemia. During the past decade, the global prevalence of cardiovascular disease has increased, reaching epidemic proportions in certain societies [8]. In the United States, CAD claims the lives of approximately 500,000 men and women each year - far greater than the number of deaths from cancer. Hypertriglyceridemia and hypercholesterolemia have been established as significant risk factors for CAD. On the other hand, high levels of HDL-C appear to be protective against CAD, whereas lower levels increase the risk for coronary artery disease [9].

The type II coronary intervention study has shown that lowering serum cholesterol and LDL-C and increasing HDL-C by drug intervention retarded the progress of CAD [10].

The frequent association of ischemic heart disease with hyperlipidemia has stimulated the search for substances that reduce blood lipid levels. In recent years, many drugs have been discovered but are reported to have adverse side effects and other disadvantages [11]. The first-line combination of drugs, cholestyramine and nicotinic acid, is the most potent, available antilipidemic agent but suffers from the drawback of bulk dosage and high incidence of side effects. The advent of the HMG-CoA reductase inhibitors or "statins" has revolutionized the treatment of dyslipidemia [12]. Though they are efficacious and well-tolerated, they are not devoid of side effects, and the high cost is a limiting factor.

Therefore, it is necessary to search for new agents that are equally effective but more economical. There are several plants described in the traditional system of medicine to have lipid-lowering properties. As the present study was an early phase II trial, it was therefore decided to carry out in uncomplicated hyperlipidemic patients with no significant history of CAD. In our study, no dietary restriction was advised. Placebo therapy was given for 14 days, and the levels estimated on day 15 were considered basal and compared.

Several clinical trials have been carried out on the efficacy of different preparations of guggulu. In the study carried out by Ballantyne CM using guggulu, the reduction of cholesterol and triglyceride levels at the end of 12 weeks of therapy was 17.5 ± 9.9% and 30.3 ± 18.4%, respectively [13]. In a similar study carried out by Jain AK et al., using garlic extract showed a 6% reduction in total cholesterol and 11% reduction in LDL-C, and there was no change seen in HDL-C [14].

The recent study showed a 20.14 ± 15.64% reduction in total cholesterol, 52.02 ± 20.04% in serum triglyceride, and 15.48 ± 28.42% reduction in LDL-C at the end of the third month (day 106). Serum HDL-C showed a rise of about 15.41 ± 26.85% at the end of the third month. The ratio of total cholesterol to HDL-C and LDL-C to HDL-C are good predictors of CAD and treatment benefits. Reduction of the ratios minimizes the consequences of atherosclerosis. TC: HDL-C and LDL-C: HDL-C ratios should be maintained at less than four and less than three, respectively. In the present study, the TC: HDL-C ratio and LDL-C: HDL-C ratio reduced to 4.39 ± 1.02 and 2.96 ± 1.001, respectively.

In our study, the drug was well tolerated and did not produce side effects like diarrhea, eructation, hiccups, restlessness, and apprehension, as reported in earlier studies [15, 16].

The side effects observed in the present study were a feeling of fullness in epigastrium, constipation, and skin rash. As drugs like β-blockers and thiazides are known to increase triglyceride levels and lower HDL-C levels, patients taking these drugs were grouped separately and compared with the other group not on these drugs. There was no significant change observed between the two groups. Out of the total 19 patients, nine patients who were on these drugs were grouped separately as group A (n=9), and the data was analyzed and compared with the other group (n=10) who were on other antihypertensive drugs.

Limitations

Firstly, the sample size of the study is small; if this had been large enough, then a better conclusion may have been derived. However, as the present study is a pilot study, a small sample size was taken, and this issue was already addressed in the Material and Methods section. Secondly, the lipid-lowering properties of the herbal drugs may be better ascertained if tested individually, rather than giving the whole combination in one single tablet.

## Conclusions

It can be concluded that the study drug (*A. sativum, T. foenum graecum, C. mukul, P. kurroa, and P. nigrum*) is completely safe and efficacious in reducing the total cholesterol, serum triglycerides, LDL-C levels, and increasing the HDL-C levels. The hypolipidemic effect starts within one month of starting the drug and persists throughout the treatment. Therefore, the study drug can be considered good, as it caused more than a 20% fall in serum cholesterol, triglyceride, and LDL-C levels. In addition, it was moderately good in causing a rise in HDL-C levels. The drug was also effective in causing a reduction in the lipid levels in patients on β-blockers.
